# Possibilities of Using Seed Meals in Control of Herbicide-Susceptible and -Resistant Biotypes of Rye Brome (*Bromus secalinus* L.) in Winter Wheat

**DOI:** 10.3390/plants11030331

**Published:** 2022-01-26

**Authors:** Elżbieta Pytlarz, Dorota Gala-Czekaj

**Affiliations:** 1 Institute of Agroecology and Plant Production, Wrocław University of Environmental and Life Sciences, Grunwaldzki Square 24A, 50-363 Wrocław, Poland; 2 Department of Agroecology and Crop Production, University of Agriculture in Kraków, Mickiewicza 21 Ave, 31-120 Kraków, Poland; dorota.gala@urk.edu.pl

**Keywords:** non-chemical weed management, rare weeds, herbicide resistance, weed control, allelopathy, winter wheat

## Abstract

Rye brome is a rare and nuisance weed in winter wheat canopies. In recent years, farmers have complained about the inadequate chemical control of this species. This study aimed to assess the effectiveness of seed meals obtained from allelopathic crops as an environmentally-friendly alternative for the control of herbicide-susceptible (S) and -resistant (R) rye brome biotypes in winter wheat. The pot experiment was conducted in a greenhouse at the Swojczyce Research and Training Station in Wrocław (Poland) to determine the impact of seed meals from: *Fagopyrum esculentum*, *Sinapis alba*, *Phacelia tanacetifolia*, *Lupinus luteus*, *Raphanus sativus* var. *oleiformis* and *Ornithopus sativus*, at 1 and 3% doses. Wheat emergence (>90%) and early growth were not affected by the presence in the soil of seed meals (only at 1% concentration) from *P. tanacetifolia* and *R. sativus*. The efficacy of these meals (reduction of aboveground biomass) at rye brome control was the same as the herbicide or higher. Seed meals from *P. tanacetifolia* reduced the emergence of the S and R biotypes by approximately 70 percentage points (p.p.) and 30 p.p., respectively, and limited the initial growth of both biotypes. Addition to soil meals from *F. esculentum* and *R. sativus* generally reduced only initial weed growth.

## 1. Introduction

*Bromus* L. is a genus belonging to the *Poaceae* family [1,2,3] and comprises about 150 species [4]. The most frequently occurring species of brome-grasses worldwide include: downy brome (*Bromus tectorum* L.), great brome (syn. ripgut brome; *B. diandrus* Roth, syn. *Anisantha diandra* (Roth) Tsvelev), meadow brome (*B. commutatus* Schrad.), rye brome (syn. cheat; *B. secalinus* L.), soft brome (*B. hordeaceus* L.), smooth brome (*B. racemosus* L.), sterile brome (*B. sterilis* L. syn. *Anisantha sterilis* (L.) Nevski) and rescue brome (*B. willdenowii* Kunth). All of these species pose a threat to arable crops as competitive weeds [1,5,6,7].

One of the most common and harmful weed species among the *Bromus* genus is rye brome. The infestation of numerous crops with *B. secalinus* can be observed on almost all continents. Rye brome is widespread in European countries such as the United Kingdom [1,7], France [2], Germany [8], Romania [9] and Poland [3,10,11,12,13,14,15]. It is a significant problem in North America [6], especially in the USA and Canada in winter wheat production areas of the Great Plains [16,17]. In the last decade, the occurrence of rye brome has been confirmed in Asia—including in Iran [18] and Taiwan [5].

Rye brome is an annual, speirochoric plant [12], which may grow in a spring or winter form; however, winter forms are more common [15]. In Poland, in the past, *B. secalinus* was a troublesome weed in winter crops [11]. Between the 1970s and the end of 20th century, it vanished from arable fields almost completely [19,20]. The regression of rye brome caused it to be classified as a rare species threatened with extinction [21]. In recent years, the weed has unexpectedly re-emerged on arable lands and poses a serious threat to crops. Nowadays, *B. secalinus* grows mostly in winter wheat and rye. It can be found sporadically in pastures and meadows, as well as in ruderal habitats such as field borders, wastelands or roadsides [9,11,19,20]. An increased occurrence of the species was observed in the southern [10,11], northern-eastern [20] and central [22] regions of Poland.

The reasons for this re-expansion of rye brome are to be found not only in the simplification of crop rotation, climate change, and the use of more selective herbicides, but also in the rapid co-evolution of the weed with winter crops and other segetal species [12,17,20]. Davies et al. [7] have pointed to the increased use of minimum tillage and the evolution of herbicide resistance as possible causes of the more frequent occurrence of rye brome in arable fields.

The increase of rye brome’s occurrence in cereals is not reflected in the registration of herbicides for its control. Currently, in Poland, in the Recommendations for the Protection of Agricultural Plants of Institute of Plant Protection [23] for the control of *B. secalinus* in cereals, only four active ingredients are registered (mesosulfuron-methyl, propoxycarbazone-sodium, pyroxsulam, sulfosulfuron). All of them have one mode of action (MoA); they are ALS inhibitors and, according to the Herbicide Resistance Action Committee (HRAC), belong to Herbicide MoA Group 2. Moss et al. [24] classify active ingredients from the group of ALS inhibitors as substances with a high risk of developing resistance. In Herbicide MoA Group 2, the number of resistant biotypes is increasing most quickly, and, of the 524 resistant biotypes, as many as 32% are biotypes resistant to active ingredients belonging to this group. Wheat (in which brome grasses occur most frequently) is the crop with the highest confirmed number of biotypes of herbicide-resistant weeds (73 unique cases) [25]. Both worldwide and in Poland, it is a staple grain. In 2019, it was cultivated over an area of 239.6 and 2.5 M ha, respectively [26].

There are currently 24 confirmed herbicide-resistant biotypes of the *Bromus* genus worldwide. Half of these have been recorded in the last 10 years. In total, 13 resistant biotypes were found in the cultivation of wheat, of which 11 showed resistance to ALS inhibitors. There are currently two confirmed unique cases of herbicide-resistant biotypes of *B. secalinus*. They are characterized by resistance to active ingredients from Herbicide MoA Group 2 (imazamox, propoxycarbazone-sodium, pyroxsulam and sulfosulfuron) [25].

A relatively small variation in the mode of action (approx. 350 chemical compounds against weeds represent 26 different MoA) results in the selection of biotypes resistant to herbicide [27]. For many years, agricultural development has been focused on maximizing productivity, but currently the need to ensure the sustainable development of agroecosystems has become the dominant concept in agriculture. One of the goals of sustainable development and the Green Deal policy is to maintain biodiversity in the agroecosystem. The withdrawal of herbicides and the limitation of their use are coherent with the pro-ecological policy that is being promoted, as is the implementation of integrated weed management, which is considered to be the most desirable concept of weed control. There are thus several aspects to the management of weeds, such as their mechanical, cultural, ecological, biological and chemical aspects and allelopathy [28,29]. The use of the allelopathic potential of crops in the form of catch crops, living mulches, or seed meals, for example, may have a positive effect on the agroecosystem, including by providing an opportunity for inhibiting weed germination and development, and stimulating the growth of crops [30,31]. In agriculture and gardening, there is a growing interest in the production and addition into the soil of seed meals from plants belonging to various botanical families, as a non-chemical method of weed control [32,33,34]. In relation to the increase in the number of rye brome biotypes resistant to herbicides and the implementation of the EU Green Deal policy, the use of seed meals to control *B. secalinus* could be an interesting alternative.

The aim of this research was (1) to evaluate the effect of seed meals on the emergence and initial development of winter wheat and herbicide-susceptible or -resistant biotypes of rye brome; (2) to assess the possibility of using seed meals to reduce weed infestation with herbicide-susceptible or -resistant biotypes of rye brome in winter wheat; (3) to compare the effectiveness of rye brome control by seed meals with herbicide spraying.

The research hypothesis assumes that the presence of seed meals in the soil will limit the emergence and initial development of herbicide-susceptible and -resistant biotypes of rye brome, and will not affect the initial development of wheat.

## 2. Results

### 2.1. Influence of Seed Meals on Winter Wheat

**Figure 1:**
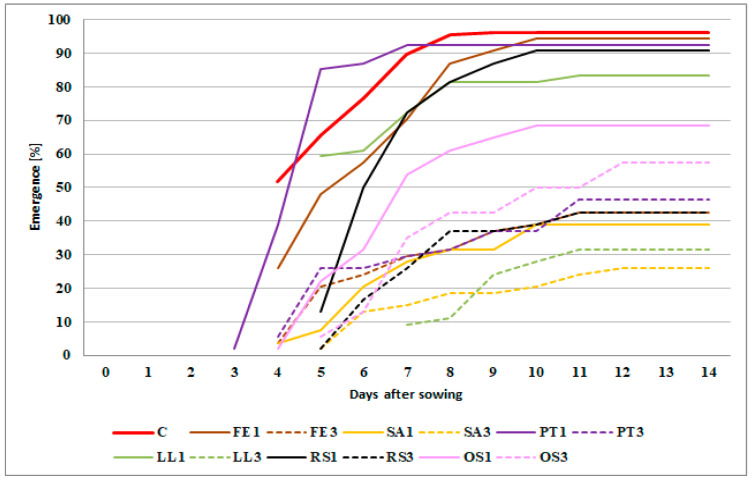
The mean emergence of winter wheat depending on type and concentration of seed meals applied. The symbols mean: C—control and seed meals from FE—*Fagophyrum esculentum*, SA—*Sinapis alba*, PT—*Phacelia tanacetifolia*, LL—*Lupinus luteus*, RS—*Raphanus sativus*, OS—*Ornithopus sativus*; 1—1% concentration of seed meals, 3—3% concentration of seed meals.

The origin and dose of the seed meal already had a clearly differentiating effect on the development of winter wheat at the emergence stage (BBCH 09) (Figure 1).

There was differentiation not only in the dynamics of emergence, but also in the number of wheat seedlings per pot. The wheat started to emerge earliest (day 3 after sowing) when treated with meal PT1; and latest (day 7 after sowing) when treated with meal LL3. The fastest rate of emergence (4 days) was found in wheat growing on the soil with meal LL1. In pots where a higher concentration of yellow lupine meal (LL3) was applied, a lengthening in the emergence of cereal shoots and a reduction in their number were observed. The fastest rate of emergence (8 days) was observed for wheat growing in the soil with the addition of FE3, SA3 and OS3 meal. The highest percentage of wheat seedlings was found in the pots without meal, i.e., in the control treatment (C)—96%. A similar percentage of emerging plants (>90%) was recorded for wheat mixed with FE1, PT1 and RS1 meals. In the case of meals of a lower concentration, the lowest percentage of grain seedlings (39%) was observed in the treatment with meal SA1. An increase in the concentration of the SA meal in the soil to 3% also resulted in the lowest percentage of seedlings among the meals tested (26%).

**Figure 2:**
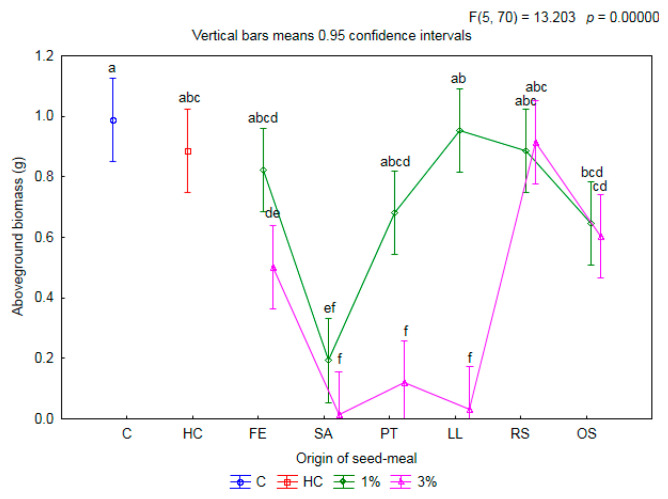
The mean aboveground biomass per one plant of winter wheat depending on origin of seed meals and their concentration. Means with various letters are significantly different, according to Tukey test (*p* ≤ 0.05). The symbols mean: C—control, HC—herbicide control, and seed meals from FE—*Fagophyrum esculentum*, SA—*Sinapis alba*, PT—*Phacelia tanacetifolia*, LL—*Lupinus luteus*, RS—*Raphanus sativus*, OS—*Ornithopus sativus*; green line—1% concentration of seed meals, rose line—3% concentration of seed meals.

The addition of seed meals to the soil also led to a modification in the aboveground biomass per one plant of winter wheat (Figure 2).

After the application of seed meals at a concentration of 1%, overall a non-significant reduction in the mass of aboveground parts of wheat was found, compared to the C (0.989 g) and HC (0.887 g) treatments. Only the SA1 meal significantly inhibited the growth of wheat biomass, by multiples of 5.1 and 4.6 respectively, compared to C and HC. In turn, the OS1 meal resulted in a significant limitation of the aboveground biomass of wheat by 34.7%, only in comparison to C. The addition to the soil of meals at a higher concentration inhibited growth of fresh mass of aboveground parts of wheat—compared both to C and HC. The exception was wheat growing on the substrate soil with the addition of RS3 meal. In this case, the aboveground biomass of wheat was on the same level as in the C and HC treatments. Moreover, the application of the OS3 meal resulted in a limitation in the aboveground biomass of wheat by 38.9%, only in comparison to C.

**Figure 3:**
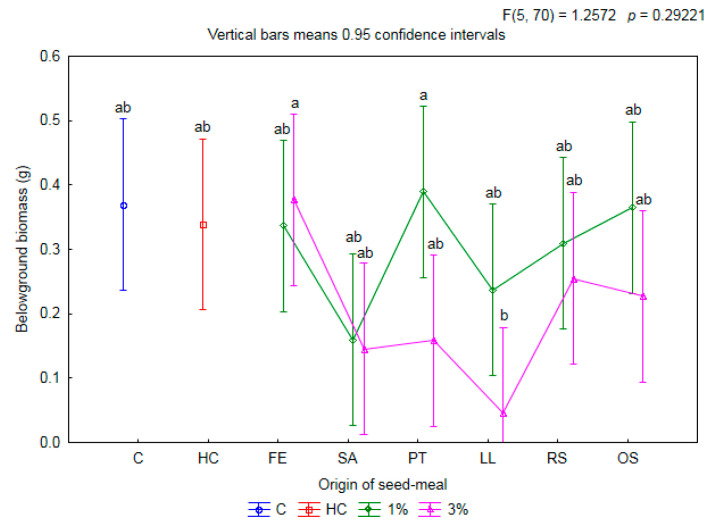
The mean belowground biomass per one plant of winter wheat depending on origin of seed meals and their concentration. Means with various letters are significantly different, according to Tukey test (*p* ≤ 0.05). The symbols mean: C—control, HC—herbicide control, and seed meals from FE—*Fagophyrum esculentum*, SA—*Sinapis alba*, PT—*Phacelia tanacetifolia*, LL—*Lupinus luteus*, RS—*Raphanus sativus*, OS—*Ornithopus sativus*; green line—1% concentration of seed meals, rose line—3% concentration of seed meals.

The type of meal and its concentration in the soil did not result in any differences in the belowground biomass per one plant of wheat compared to C and HC (Figure 3). Root biomass was found to be significantly lower only after application of the LL3 meal compared to FE1 and PT1—by multiples of 7.4 and 8.5, respectively.

**Figure 4:**
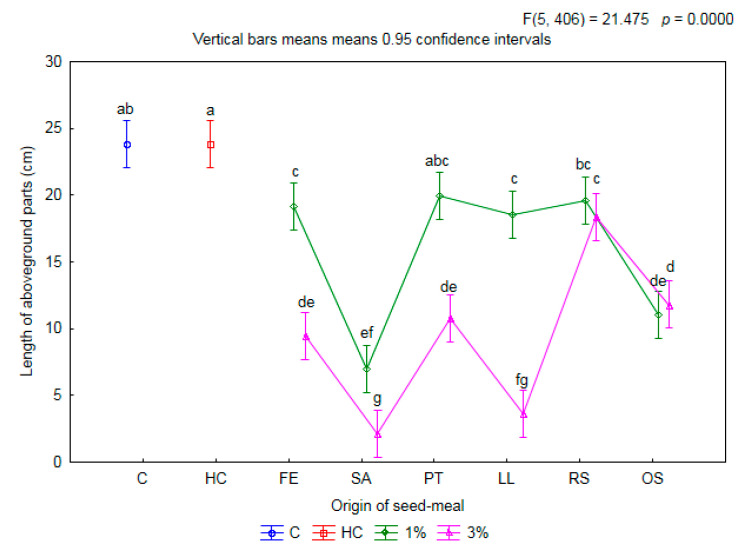
The mean length of aboveground parts of winter wheat depending on origin of seed meals and their concentration. Means with various letters are significantly different, according to Tukey test (*p* ≤ 0.05). The symbols mean: C—control, HC—herbicide control, and seed meals from FE—*Fagophyrum esculentum*, SA—*Sinapis alba*, PT—*Phacelia tanacetifolia*, LL—*Lupinus luteus*, RS—*Raphanus sativus*, OS—*Ornithopus sativus*; green line—1% concentration of seed meals, rose line—3% concentration of seed meals.

The addition to the soil of seed meals from various crop species in differing concentrations had an impact on the average length of aboveground parts of the winter wheat plants (Figure 4).

In the majority of cases, the addition of meals led to a significant limitation of up to several centimeters in the length of the aboveground parts of the wheat. After the application of meals at a lower concentration (1%), less of an inhibitory impact on the tested parameter was found overall. On average, compared to treatment C, the inhibition of growth was 41%. Only the PT1 meal enabled the length of the aboveground parts of wheat to be maintained at the same level as in the C and HC treatments, while RS1 enabled it to be maintained at the level of C. It is worth emphasizing that in treatments with the same meals (PT1 and RS1), an emergence of wheat at the level of >90% (cf. Figure 1) was observed, as well as a non-significant limitation of the fresh mass of aboveground parts of the tested crop (cf. Figure 2). The shortest (7.0 cm) parts were found in wheat growing on the soil mixed with meal SA1. An increase in the concentration of the meals applied led to a significant limitation in the length of wheat overall. SA3 was found to have the most inhibitory effect on the increase in the length of aboveground parts of wheat (length 2.1 cm). After the application of RS and OS meals, the assessed parameter remained at the same level at both concentrations.

**Figure 5:**
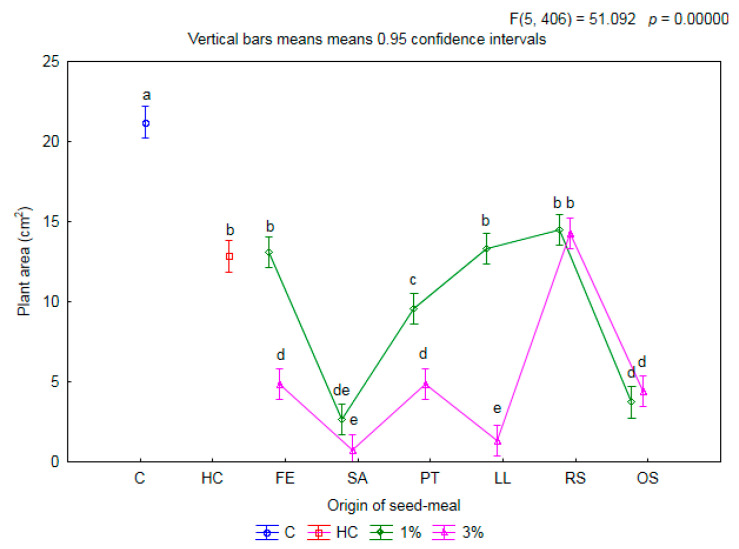
The mean plant area of winter wheat depending on origin of seed meals and their concentration. Means with various letters are significantly different, according to Tukey test (*p* ≤ 0.05). The symbols mean: C—control, HC—herbicide control, and seed meals from FE—*Fagophyrum esculentum*, SA—*Sinapis alba*, PT—*Phacelia tanacetifolia*, LL—*Lupinus luteus*, RS—*Raphanus sativus*, OS—*Ornithopus sativus*; green line—1% concentration of seed meals, rose line—3% concentration of seed meals.

Meals from tested species of donor plants at a concentration of 1% and the application of herbicide (HC) significantly limited (by 11 cm^2^ on average) development in the aboveground area of wheat compared to treatment C (21.2 cm^2^) (Figure 5). In the case of meals FE1, LL1 and RS1, no decrease in the aboveground area of wheat was found compared to the treatment HC. Interestingly, these same meals (FE1, LL1, RS1) also did not cause any significant decrease in the mass of aboveground parts (cf. Figure 2) or in the mass of belowground parts (cf. Figure 3) of the wheat tested, compared both to HC and to C. The reduction in emergence was 13 percentage points (p.p.) at most for LL1 (cf. Figure 1). After application at a higher concentration, a further decrease in the area of the aboveground parts of the wheat (by 4 cm^2^ on average) was found overall, compared to the treatments C and HC. Only the addition to the soil of the RS3 meal did not limit the aboveground area of wheat, compared to HC. The application to the soil of this meal also did not result in a reduction in the aboveground biomass of wheat (cf. Figure 3). This may be evidence of the neutral impact of this meal on the tested crop variety.

### 2.2. Effectiveness of Seed Meals in Reduction of Rye Brome Growth

**Figure 6:**
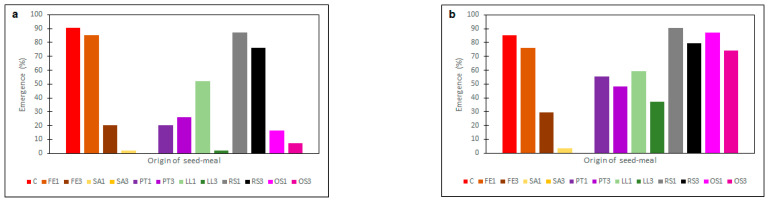
The mean emergence of herbicide-susceptible (**a**) and -resistant (**b**) biotypes of rye brome depending on origin of seed meals and their concentration (14 days after sowing). The symbols mean: C—control and seed meals from: FE—*Fagophyrum esculentum*, SA—*Sinapis alba*, PT—*Phacelia tanacetifolia*, LL—*Lupinus luteus*, RS—*Raphanus sativus*, OS—*Ornithopus sativus*; 1—1% concentration of seed meals, 3—3% concentration of seed meals.

The type and dose of meal added to the soil led to differences in the number of seedlings of rye brome of both the susceptible (Figure 6a) and the resistant (Figure 6b) biotype. The emergence of seedlings of the herbicide-susceptible biotype was inhibited most weakly on the soil with the addition of FE1 and RS1 and RS3 meals. The percentages of seedling emergence were 85%, 87% and 76% respectively. Independently of the biotype, the lowest percentage of seedling emergence was recorded after the application of the SA meal (2% or lack of seedling emergence). It should, however, be noted that these meals also significantly limited the development of the wheat (cf. Figure 1, Figure 2, Figure 3, Figure 4 and Figure 5). For this reason, the use of SA meals in the cultivation of wheat to limit the development of rye brome may be of limited significance.

Moreover, in the resistant biotype, a weaker reaction of emerging seedlings to the applied meals was observed in comparison to the susceptible biotype. The FE3 meal was fairly effective at limiting emergence (along with the SA meal). After the application of this meal, the percentage of seedling emergence for the resistant biotype of rye brome was 30%. With an increase in the concentration of the meals, an increase in the limitation of seedling emergence was observed, although the difference between the concentrations was not as big as in the case of the herbicide-susceptible biotype.

**Figure 7:**
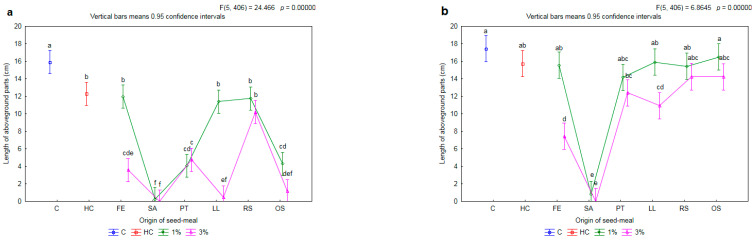
The mean length of aboveground parts of herbicide-susceptible (**a**) and -resistant (**b**) biotypes of rye brome depending on origin of seed meals and their concentration. Means with various letters are significantly different, according to Tukey test (*p* ≤ 0.05). The symbols means: C—control, HC—herbicide control, and seed meals from: FE—*Fagophyrum esculentum*, SA—*Sinapis alba*, PT—*Phacelia tanacetifolia*, LL—*Lupinus luteus*, RS—*Raphanus sativus*, OS—*Ornithopus sativus*; green line—1% concentration of seed meals, rose line—3% concentration of seed meals.

Application of herbicide resulted in a limitation in the length of aboveground parts of the herbicide-susceptible biotype of rye brome by 23% compared to C (15.9 cm) (Figure 7a).

In each case, the assessed parameter was found to have decreased in length after addition of the meal. The application of meals FE1, LL1 and RS1 allowed the weed plants to be shortened to the same level as with the spraying of herbicide (HC), i.e., by 26% on average, compared to C. This effect increased after the addition of the meals OS1, PT1 and SA1. It is worth underlining that the addition of the meals PT1 and RS1 did not have an impact on the length of the aboveground parts of the wheat (cf. Figure 4). For the FE and LL meals only, an increase in concentration from 1% to 3% caused a significant increase in the inhibition of the development of the length of the aboveground parts of the susceptible biotype of rye brome—by 70% and 96%, respectively.

There were no differences in the length of aboveground parts of the resistant biotype of rye brome as a result of the application of herbicide and the majority of meals at a concentration of 1% compared to C (17.4 cm) (Figure 7b). The exception was the meal SA1. It limited the tested parameter by 95% compared to C. Similarly, as in the case of the susceptible biotype of rye brome, it was only after an increase in the concentration of FE and LL meals from 1% to 3% that there was found to be a further decrease in the length of the aboveground parts of the weed; this decrease was by 52% and 31%, respectively.

**Figure 8:**
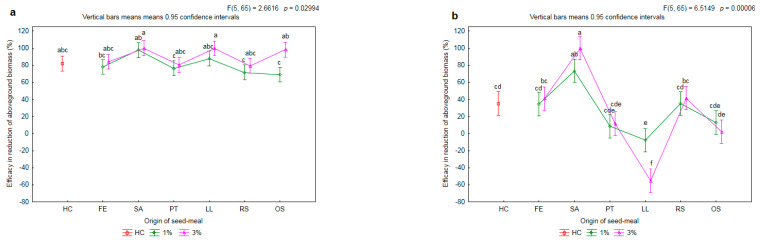
The mean efficacy in reduction of aboveground biomass of herbicide-susceptible (**a**) and -resistance (**b**) biotypes of rye brome depending on origin of seed meals and their concentration. Means with various letters are significantly different, according to Tukey test (*p* ≤ 0.05). The symbols mean: C—control, HC—herbicide control, and seed meals from: FE—*Fagophyrum esculentum*, SA—*Sinapis alba*, PT—*Phacelia tanacetifolia*, LL—*Lupinus luteus*, RS—*Raphanus sativus*, OS—*Ornithopus sativus*; green line—1% concentration of seed meals, rose line—3% concentration of seed meals.

The efficacy of the tested meals in the reduction of the aboveground biomass of the herbicide-susceptible biotype of rye brome was on the same level as the efficacy with the herbicide treatment (HC) (Figure 8a). On average, it was 80%. The application of the meals LL3, OS3, SA1 and SA3 resulted in a limitation on the biomass of the aboveground parts of over 95%. There was found to be a significant increase, by 29 p.p., in efficacy together with an increase in the concentration of meal only for the OS meal.

There were differences in the effectiveness of limitation of the biomass of aboveground parts of the herbicide-resistant biotype of rye brome after the application of seed meals (Figure 8b). In the weeds growing on the soil with the addition of the FE1, PT1, RS1 and OS1 meals, the efficacy was found to be on the same level as with the chemical control, i.e., approximately 35%. Moreover, the application of the aforementioned meals did not cause any significant decrease in the biomass of the aboveground parts of the wheat in relation to HC (cf. Figure 2). The SA1 meal caused an increase in efficacy by 38 p.p. with reference to HC. In turn, the LL1 meal caused an increase in the biomass of aboveground parts of the weed by 8 p.p. in relation to C. Together with an increase the concentration of the LL meal to 3%, a further decrease in the efficacy of reduction of aboveground biomass by 47 p.p. was observed.

**Figure 9:**
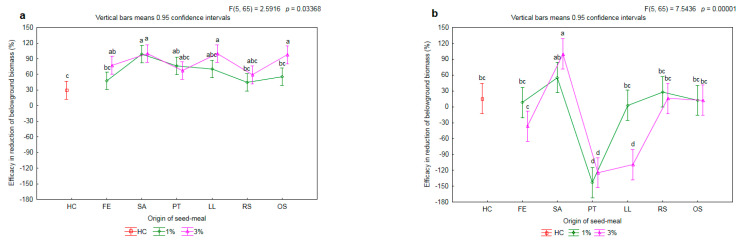
The mean efficacy in reduction of belowground biomass of herbicide-susceptible (**a**) and -resistance (**b**) biotypes of rye brome depending on origin of seed meals and their concentration. Means with various letters are significantly different, according to Tukey test (*p* ≤ 0.05). The symbols mean: C—control, HC—herbicide control, and seed meals from: FE—*Fagophyrum esculentum*, SA—*Sinapis alba*, PT—*Phacelia tanacetifolia*, LL—*Lupinus luteus*, RS—*Raphanus sativus*, OS—*Ornithopus sativus*; green line—1% concentration of seed meals, rose line—3% concentration of seed meals.

The application to the soil of selected meals—namely SA and PT at a concentration of 1% caused a significant increase in efficacy in reduction of belowground biomass of the susceptible biotype of rye brome in relation to the application of herbicide (HC; 29.5%) (Figure 9a). 

The increase in the efficacy of the aforementioned meals compared to the HC treatment amounted to 70 and 47 p.p. respectively. A significant increase in the efficacy of the meal (by 42 p.p.) was found, together with an increase in its concentration in the soil for the OS meal only. Moreover, the meals FE3, SA3, LL3 and OS3 significantly limited the development of the belowground biomass of the weed with reference to HC. The increase in the efficacy of the reduction in the growth in mass amounted to: 48, 71, 70 and 68 p.p. respectively.

The application of meals from tested donor plants had a varying impact on the development of biomass of belowground parts of the herbicide-resistant biotype of rye brome (Figure 9b). After the application of nearly all the meals at a concentration of 1% (FE, LL, RS, OS), a reduction in the fresh mass of the aboveground parts of a level comparable to that seen after spraying with herbicide (HC; 12%) was observed. In the case of the SA1 meal, the efficacy was found to be over three times higher (55%) than after the application of herbicide. It should be emphasized that the SA1 meal also resulted in a significant limitation in the growth of the mass of the aboveground parts of the herbicide-susceptible biotype of rye brome compared to HC (cf. Figure 9a). In the case of the application of the SA meal at a higher concentration (3%), a significant increase, in relation to HC, was observed in the efficacy of the reduction of belowground biomass (by 85 p.p.). A significant increase in the efficacy of the meal was found, together with an increase in its concentration in the soil, only for the LL meal. It is worth underlining that, in the case of wheat (cf. Figure 3), the application of meals did not lead to any differences in its belowground biomass.

## 3. Discussion

On cereal fields, it is especially difficult to control monocotyledonous weed species, including those from the *Bromus* genus, which are also highly competitive with crop plants [35,36]. The chemical control of brome grasses has been the focus of much research worldwide. However, an effective and dependable solution is still to be found [6,16,17,37]. A significant problem in the management of *Bromus* spp. is the occurrence and evolution of herbicide resistance [7,38,39], as a consequence of the limited rotation of herbicides, as well as the application of simplifications to crop rotations and monocultures [40,41,42]. Additionally, herbicides pose toxicological and ecological threats, especially toward non-target organisms [43].

There are numerous works in the international body of research concerning non-chemical methods of control of brome-grasses [44,45,46]. Their authors show that rhizobacteria can be used in the biocontrol of *Bromus* spp., including—*B. secalinus* [47]—one of the most widespread and damaging weeds of the *Bromus* genus on a global scale [1,2,6,7,8,15]. Some authors point out that using crop rotation reduces the density of rye brome panicles per unit area, but does not eliminate it entirely [17]. Stone at al. [17], show that the rotation out of winter wheat for one growing season in comparison to continuous cropping winter wheat reduced up to 87% rye brome panicles. In non-chemical weed control, biological methods play a significant role alongside cultural methods, whereas suppressing weeds by using the allelopathic phenomenon is considered to be one of the most innovative methods of weed control [48,49]. Different agronomic methods enable the practical utilization of allelopathic plants in the form of seed meals [32,33,34,50].

Based on our own experiments, meal from white mustard (*Sinapis alba*; SA) proved to be the most effective at inhibiting initial growth of herbicide-susceptible and herbicide-resistant biotypes of rye brome. After its application, the percentage of germinating seeds of *B. secalinus* was 3.7% at most, and the average length of the aboveground parts of plants that emerged was less than 1 cm. Our results are consistent with the work of many authors [32,34,51], who also underline the strong inhibitory action of meal from *S. alba* in relation to weeds. These authors show that seed meals from white mustard reduced the growing seedlings of the following weeds: monocotyledonous (*Poa annua* L., *Digitaria ischaemum* (Schreb.), *Panicum dichotomiflorum* Michx.), dicotyledonous (*Stellaria media* L., *Oxalis corniculata*, *Physalis angulata* L., *Amaranthus spinosus* L., *Cyperus esculentus* L.), as well as liverwort (*Marchantia polymorpha* L.). In our research, the reduction in the aboveground biomass of the herbicide-susceptible biotype after application of the SA meal was approximately 98–100% and, in the case of the herbicide-resistant biotype, it was 73–100% (for concentrations of 1 and 3%, respectively). Dastgheib et al. [6] show that a mixture of terbutryn + terbuthylazine applied at the tillering stage of wheat results in a similar level of efficacy, with more than a 90% reduction in ripgut brome (*Bromus diandrus* Roth) biomass. In our study, the efficacy of herbicide with propoxycarbazone-sodium in aboveground biomass reduction was 35–82% for the resistant and susceptible biotype of rye brome, respectively. Unfortunately, in our research, SA meal also had an inhibiting effect on the initial development of winter wheat—the crop in which *B. secalinus* occurs most frequently [10,13,17,37]. In relation to this, its use as a biological herbicide in the cultivation of winter wheat is impossible. Our finding is supported by [32,33,34], who also confirm the inhibitory action of *S. alba* seed meal on the growth of various species of crops: maize (plant number and biomass), cucurbits (severely reduced yield) and lettuce (emergence).

This study revealed that meal from lacy phacelia (*Phacelia tanacetifolia*; PT) and from fodder radish (*Raphanus sativus* var. *oleiformis*; RS) at a concentration of 1% limited the development of the aboveground biomass of the herbicide-susceptible biotype of rye brome in the same way as spraying with herbicide. Importantly, in our research, meals from PT1 and RS1 did not result in any significant limitation of germination and development in the mass of aboveground parts of winter wheat compared to the control (C) and the herbicide control (HC). Partly similar results were obtained by Pużyńska et al. [32]. The authors assessed the impact of seed meal from wild radish (*Raphanus raphanistrum* L.) on maize (*Zea mays* L.) and two weed species—barnyard grass (*Echinochloa crus-galli* (L.) P.Beauv.) and redroot pigweed (*Amaranthus retroflexus* L.). As in our experiment, meal from wild radish was not found to have any significant impact on the dry mass of maize shoots. Pużyńska at al. [32] show that meal from wild radish also did not impact on the dry mass of aboveground parts of barnyard grass and redroot pigweed. Many authors draw attention to the allelopathic properties of lacy phacelia and the possibility of its use as a natural product in non-chemical weed management (weeds monocotyledonous: *Sorghum halepense* (L.) Per., *E. crus-galli*, weeds dicotyledonous: *Portulaca oleracea* L., *Chenopodium album* L., *Solanum nigrum* L., *A. retroflexus*, *Convolvulus arvensis* L., *Tribulus terrestris* L., *Sisymbrium officinale* (L.) Scop.) [52,53,54,55,56]. The situation is similar with buckwheat. Most authors focus, however, on the potential for the limitation of the development of weeds by root secretions from this species [57,58,59,60]. Our own studies found the emergence of both biotypes of rye grass to be strongly limited after the addition of a higher dose of meal into the soil. The application of this dose of seed meal from buckwheat also resulted in a significant limitation in the length of the aboveground parts of both the weed and the wheat. In studies by Mioduszewska et al. [61], a limitation on the initial development of wheat after the application of extract from the aboveground parts of buckwheat was also found.

## 4. Materials and Methods

### 4.1. Plant Materials

In the experiment, two acceptor species were tested. The first was the crop, common wheat (*Triticum aestivum* L. cv. ‘Agil’) and the second was the weed, rye brome (*B. secalinus*). Winter wheat seeds were certified and marked as a degree C/1 (certified seed from the first multiplication, obtained after one multiplication of the basic seed). The herbicide-susceptible (S) and -resistant (R) biotypes of rye brome were harvested from winter wheat fields in July 2020. The characteristics of both the S and R biotypes of rye brome are presented in Table 1. The resistant biotype of weed was characterized by a low resistance index (2 ≤ R ≤ 4) to propoxycarbazone-sodium.

### 4.2. Seed Meals and Their Preparation

Qualified seeds of selected crop species (Table 2) were milled the day before the pot experiment was started. All the collected commercial seeds were grounded to meals in a Fritsch Pulverisette 11 laboratory mill (Idar-Oberstein, Germany).

### 4.3. Herbicide Characteristics

The active ingredient of the herbicide used in the experiment is propoxycarbazone-sodium (70%). According to the HRAC classification, it is classified as belonging to Herbicide MoA Group 2. The active ingredient presents a systemic type of action. Recommended application per leaves. It is a selective herbicide and has the formulation of water-soluble granules (SG).

### 4.4. Soil Characteristics

A soil that was used in the experiment was formed from light loamy sand underlaid with poorly loamy sand and was classified as IVb quality class (in Poland, equivalent to good rye complex). The topsoil (0–30 cm) was characterized by the following parameters: pH_KCl_ 5.82; P 86.4; K 27.5; Mg 131.0 (mg·kg^−1^ of soil) and C_org_ 0.41%. The soil was taken after harvesting the forecrop of organic forage pea cv. ‘Roch’.

### 4.5. Set-up and Management of Pot Experiments

Two series of pot experiments were carried out during 2020 and 2021. Series I began in November and series II started in March, in a greenhouse at Wrocław University of Environmental and Life Science’s Research and Training Station in Swojczyce (Wrocław, Poland). In both, the lighting and thermal conditions were regulated. The first experimental factor was the type of seed meal; the second was the dose of seed meal. Each acceptor (winter wheat and herbicide-susceptible and -resistant biotypes of rye brome) was analyzed individually.

Before starting the experiment, the soil was sieved over 1 cm mesh screens to rid the soil of post-harvest residue. The experiment was set up as a totally randomized design with three pots as replications. Production pots 0.5 L in volume were filled up with a mixture of 500 g of soil and one of the seed meals in an amount of 1 or 3% (*w/w*), separately. The control (C) and herbicide control (HC) pots did not contain any addition of meals. Nine grains each of either winter wheat or either of the biotypes of rye brome (S or R) were sown into soil-filled pots. Fourteen days after sowing, the number of plants per pot was equalized to five if the number of seedlings allowed. The HC treatment was sprayed on the two leaves of unfolded-stage (BBCH 12) rye brome in the spray chamber (APORO Sp. z o.o., Poznań, Poland). The dose of propoxycarbazone-sodium was 56 g ha^−1^·200 L of water. Experiments were harvested when the plants of winter wheat in the C treatment were at the four leaves unfolded stage (BBCH 14).

### 4.6. Measurement Range

#### 4.6.1. Winter Wheat

Wheat emergence was counted daily for 14 days after sowing. At the end of each series, the plants were pulled out and counted. The fresh weight of aboveground and belowground parts was determined (roots were washed and dried on a paper towel) using a WTC 2000 scale from RADWAG (Cracow, Poland). The length of the aboveground parts was measured. The next day, the area of the aboveground parts of wheat was measured with using a CI-202 LASER LEAF AREA METER from CID Bio-Science (Camas, WA, USA).

#### 4.6.2. Rye Brome

During the harvest, the plants were pulled out and counted. The fresh weight of above and belowground parts (roots were washed and dried on a paper towel) was determined using a WTC 2000 scale from RADWAG (Cracow, Poland). On this basis, the efficacy of biomass reduction of the tested treatments in relation to the control treatment (C) was calculated. A minus value of the index indicates an increase in the mass of rye brome with the applied seed meals. The length of aboveground parts of the weeds were also measured.

### 4.7. Statistical Analysis

Statistical analysis was carried out with using the two-way variance analysis (type of seed meal and dose of seed meal), using Statistica 13.3 software (TIBCO Software Inc., Tulsa, OK, USA). In order to check the normality of the distribution, the Shapiro–Wilk test was performed. The homogeneity of variance was checked using the Levene test. In order to determine and verify the relationships, Tukey’s post-hoc test was performed with a significance level of *p* ≤ 0.05.

## 5. Conclusions

The study found that selected seed meals can constitute an alternative to herbicide management strategies for the control of herbicide-susceptible and -resistant (to propoxycarbazone-sodium) biotypes of rye brome in winter wheat. Wheat emergence and initial growth were not affected by the presence of seed meals from common buckwheat (*Fagopyrum esculentum*), lacy phacelia (*Phacelia tanacetifolia*) and fodder radish (*Raphanus sativus* var. *oleiformis*) at 1% concentrations in the soil. The efficacy of these seed meals at the control of rye brome was at the same level as the herbicide or higher. An increase in the concentration of seed meals is not recommended due to the reduction in wheat emergence. Seed meals obtained from lacy phacelia reduced the emergence and initial growth of both biotypes of weeds, but seed meals from common buckwheat and fodder radish limited only the initial weed growth. Furthermore, despite the high efficacy of seed meals from white mustard at reducing emergence of rye brome, they are not recommended for the control of herbicide-susceptible and resistant biotypes of rye brome due to their inhibition of wheat growth. Future experiments should focus on a more comprehensive examination of seed meals in weed management by taking other herbicides, another level of herbicide resistance and other species of crops or weeds.

## Figures and Tables

**Table 1:** Characteristics of herbicide-susceptible (S) and -resistant (R) biotypes of rye brome (*Bromus secalinus* L.) used in the pot experiments. ED50 values express the effective dose of propoxycarbazone-sodium (HRAC 2) causing a 50% reduction in plant biomass (ED50).

Biotype	ED50 (g ha^−1^)	Site (Coordinate)
**S**	16.26	Wrocław (51.132663 N 17.117230 E)
**R**	48.86	Wielowieś (51.339435 N 16.373906 E)

**Table 2:** Crop species and cultivars used to prepare the seed meals.

Name		Cultivar	Abbreviation
English	Latin		
Common buckwheat	*Fagopyrum esculentum* Moench.	Panda	FE
White mustard	*Sinapis alba* L.	Bardena	SA
Lacy phacelia	*Phacelia tanacetifolia* Benth.	Anabela	PT
Yellow lupin	*Lupinus luteus* L.	Mister	LL
Fodder radish	*Raphanus sativus* L. var. *oleiformis* Pers.	Adagio	RS
Common birdsfoot	*Ornithopus sativus* Brot.	Bydgoska ^1^	OS

^1^ Variety not included in the national register.

## Data Availability

Not applicable.
